# Analysis on spatial difference and spillover effect of development resilience index of sports industry: A case study of 285 cities in China

**DOI:** 10.1371/journal.pone.0295313

**Published:** 2023-12-01

**Authors:** Yihao Li, Yue Yuan, Na Cheng

**Affiliations:** 1 General Graduate School, Dongshin University, Naju, JeollaNamdo, South Korea; 2 Jilin Institute of Physical Education, Changchun, Jilin, China; Southern Taiwan University of Science and Technology, TAIWAN

## Abstract

The sustainable development of the sports industry has garnered extensive attention worldwide. In this study, after a rigorous explanation of the connotation of the sports industry development resilience coefficient (SIDRC), the Topsis model and exploratory spatial data analysis were comprehensively employed to evaluate and visualize the SIDRC of 285 cities in China. Additionally, a spatial econometric model was constructed to explore the influencing factors of SIDRC. The major conclusions drawn from this study are as follow: (1) While the SIDRC has improved significantly over the study period, it still remains overall at a low level of resilience with a widening gap between cities. (2) A strong spatial imbalance exists in the distribution of SIDRC, with coastal regions demonstrating greater resilience compared to the central and western regions, and provincial capital cities faring better than other cities. (3) Policy support index, economic development level, structural diversity of the sports industry, and social participation play crucial roles in promoting SIDRC. Finally, social participation has a positive impact on SIDRC in neighboring cities by facilitating resource sharing, market expansion, and extending the industrial chain. The paper concludes by offering recommendations such as increasing the construction of sports markets and public participation, which can optimize the layout of the sports industry and enhance industrial development resilience.

## 1. Introduction

The sports industry as an emerging industry has vigorous market vitality and potential economic growth space [1]. As people pursue a healthy lifestyle and with the gradual release of post-pandemic consumption dividends, countries around the world have begun to regard the sports industry as an important pillar industry to promote economic development [2]. By the end of 2021, the total value of China’s sports industry had reached 2,948.3 billion yuan, an increase of 7.621% compared to 2018. The proportion of the output value of sports goods manufacturing industry continued to rise, the export value of sports goods exceeded 290 billion yuan, and the scale of online sports users was close to 397 million. In addition, according to the relevant data released by the National Bureau of Statistics, by the end of 2022, the number of sports venues in China reached 4.227 million, an increase of 6.445% compared to 2021. The per capita sports venue area increased from 2.410 square meters to 2.621 square meters. Similarly, developed countries and regions such as the United States, the European Union, Japan, and South Korea have also achieved significant results in the development of the sports industry and have formed relatively standardized and sustainable industrial systems. For example, the NFL, MLB, NHL, and NPB have strong influence in the Asian and global sports markets and have broad market prospects. In comparison, although China’s sports industry has shown a steady and continuous growth trend, it is still in the initial stage of building a commercialized, market-oriented, and large-scale sports industry ecosystem [3].

The sports industry, as a new type of economic activity, can roughly be divided into three aspects: the production and sale of sports equipment, infrastructure construction, and the sports service industry, which are deeply rooted in the complex system composed of human society, culture, and economy [4, 5]. Existing research usually examines the development of the sports industry from an economic perspective, including the impact mechanism of sports activities on the economy, the evaluation of policy effects on the sports industry, and the balance between competitive and commercial sports [5]. These studies provide a macroscopic view of the development of the sports industry and have a positive effect on optimizing the industry’s layout. Additionally, the introduction of concepts such as competitiveness and high quality provides new ideas and methods for evaluating the sports industry, shifting research perspectives from qualitative analysis to quantitative evaluation. This transformation not only enriches the development theory of the sports industry but also brings broader research space [6, 7]. For example, predictions of the trend in the development of the sports industry, differences in the sports industry between regions and their influencing factors, etc. In combination with the development experience of countries such as the EU and the USA, the commercialization and marketization of the sports industry is a future trend. With more and more brands and companies entering the sports industry, how to manage it as a profitable business has become an unavoidable realistic problem [8–10]. Therefore, the concepts of sports management and marketing have emerged, focusing on the management and marketing strategies of sports organizations [11, 12], such as brand building, sponsorship relationships, event arrangements, etc. Under the background of digital transformation, technologies such as big data, artificial intelligence, and the Internet of Things have played a key role in sports data analysis [13, 14], game strategy optimization, team performance evaluation, etc. At the same time, it has also given birth to new sports such as e-sports, which undoubtedly will bring new opportunities and challenges for the development and digital transformation of the sports industry [15]. The deep coupling of the sports industry with society and culture is also one of the research hotspots, such as the impact of sports events on the city image [16], the creation and transformation of sports culture, etc.

In summary, existing studies usually discuss the development model and marketing strategy of sports enterprises from the perspective of economics or management. Although these studies have certain practical significance to reveal the development law of sports industry and promote the sustainable development of the industry, there are still the following shortcomings: (1) the research field is scattered. Existing scholars usually discuss the development trends and optimization paths of the sports industry from their own fields, leading to relatively scattered research content and methods, which makes it difficult to form a comprehensive understanding of the development of the sports industry; (2) qualitative analysis is the main research method. The lack of quantitative analysis methods is a major characteristic of existing research, which should be given more attention in future research. Introducing quantitative methods or models into the evaluation of sports industry development can effectively reduce the impact of subjectivity on research results; (3) The research scale is single. The existing research usually focuses on the micro-scale research, but pays little attention to the macro-scale evaluation method and index system of industrial development resilience.

In view of the problems existing in the current research, this study takes 285 cities in China as the research object, based on theoretical and ideological fields such as economic geography, spatial economics, and system science, comprehensively using Topsis model and exploratory spatial data analysis to evaluate the resilience of the sports industry development, identify its differences in the time and space dimensions, and construct spatial econometric models to discuss the impact factors and spatial spillover effects of sports industry development resilience index. The main contributions of this study include: (1) proposing the concept of industry development resilience index. The coupling and complexity of the sports industry determine that a single-dimensional evaluation is one-sided. Compared with evaluation indicators such as competitiveness and high quality, development resilience emphasizes the dynamism, comprehensiveness, and adaptability of the sports industry, which has richer connotations; (2) constructing a quantitative evaluation system of indicators. This study constructs an evaluation system of sports industry development resilience from four dimensions: input and output, structure and innovation, market and consumption, and society and policy, which may provide reference for subsequent related research; (3) comparing the gap in regional sports industry development. Using spatial correlation indices to reveal the agglomeration features of the sports industry in space, and identifying the leapfrog of sports industry development resilience index at different time points; (4) discussing the spatial spillover effects of SIDRC. By constructing spatial econometric models to identify the impact factors of sports industry development resilience index and the degree and direction of impact on adjacent cities. Recognizing the gap between regions and spatial organizing patterns may provide empirical evidence for relevant departments to plan sports industry layout and enhance industry development resilience, and also provide a Chinese template for the development of sports industry in developing countries.

## 2. Methods and data

### 2.1. Topsis evaluation model based on entropy weight method

The TOPSIS model is a method for ranking the superiority and inferiority of evaluation objects based on the proximity degree to the idealized target [17, 18]. Its essence is a distance comprehensive evaluation method. By assuming positive and negative ideal solutions, the distance between each evaluation indicator and the positive and negative ideal solutions are respectively calculated to determine the closeness between the evaluation object and the ideal sample, and thus judge the superiority and inferiority of each evaluation object. This method has good applicability in the evaluation of the resilience of the sports industry development involving a large number of indicators. The specific steps are as follows:

***Step 1*:** Indicator normalization and standardization. Drawing on existing literature, the entropy weight method is used to assign weights to each indicator, which can avoid subjective factors affecting indicator preferences. Based on this, the weight vector *W* = (*w*_1_, *w*_2_, …, *w*_*m*_) and the normalized matrix are obtained.
***Step 2*:** Calculate the weighted normalized matrix.
$$Z={\left(z_{ij}\right)}_{n\times m}=(p_{ij}\times w_{j})$$ (1)
***Step 3*:** Determine the positive and negative ideal solutions. The positive ideal solution refers to the sample with the highest resilience in the development of the sports industry, where all indicators have reached their optimal values. Similarly, the negative ideal solution refers to the sample with the lowest resilience in the development of the sports industry, where all indicators have the worst values.
***Step 4*:** Calculate the distance between each evaluation object and the positive and negative ideal solutions, where *i* − 1, 2, …, *n*.
$$D_{i}^{+}=\sqrt{\sum_{j=1}^{m}{\left(z_{ij}-z_{j}^{+}\right)}^{2}}$$ (2)
$$D_{i}^{\text{-}}=\sqrt{\sum_{j=1}^{m}{\left(z_{ij}-z_{j}^{\text{-}}\right)}^{2}}$$ (3)
***Step 5*:** Calculate the resilience index of the sports industry development for each city. The calculation result is in the range of [0,1], and the closer it is to 1, the closer the evaluation object is to the ideal solution.
$$SIDRC_{i}=\frac{D_{i}^{-}}{D_{i}^{+}+D_{i}^{-}}$$ (4)

### 2.2 Exploratory spatial data analysis

Global spatial autocorrelation and local spatial autocorrelation are the two most commonly used methods in exploratory spatial data analysis. They are characterized by directly exploring hidden relationships, patterns, or trends in the data without the need for prior theoretical or hypothetical assumptions, while also revealing deviations from common models that are unpredictable [19].

**(1) Global spatial autocorrelation.** Global spatial autocorrelation is mainly used to determine the degree of spatial aggregation of the resilience index of the sports industry, which checks whether there is a spatial clustering trend [20, 21]. It is usually represented by *Moran’s I*. The formula is as follows:

$$Moran’sI=\frac{\sum_{i=1}^{n}\sum_{j=1}^{n}w_{ij}(X_{i}-\bar{X})(X_{j}-\bar{X})}{S^{2}\sum_{i=1}^{n}\sum_{j=1}^{n}w_{ij}}$$ (5)

$$S_{2}=\frac{1}{n}\sum_{i=1}^{n}{\left(X_{i}-\bar{X}\right)}^{2}$$ (6)

$$\bar{\underset{˙}{X}}=\frac{\sum_{i=1}^{n}X_{i}}{n}$$ (7)

where *X*_*i*_ represents the SIDRC of city *i*, n represents the sample size, and *W*_*ij*_ represents the inverse distance space weight matrix. The range of the global *Moran’s I* is [–1,1]. When *moran’sI* > 0, it indicates a positive spatial correlation of SIDRC, and the closer the value is to 1, the more areas tend to spatial clustering with similar SIDRC. When *moran’sI* < 0, it indicates a negative spatial correlation of SIDRC, and the closer the value is to -1, the more areas tend to diffuse with similar SIDRC. When *moran’sI* = 0, it means that SIDRC is randomly distributed in geographical space.

**(2) Local spatial autocorrelation.** The local spatial autocorrelation reveals the specific spatial correlation pattern of SIDRC, which focuses more on local changes compared to global spatial autocorrelation. The formula for calculating local *Moran’s I* is as follows:

$$L_{i}=\frac{\sum_{i=1}^{n}W_{ij}(X_{i}-\bar{X})(X_{j}-\bar{X})}{S^{2}}$$ (8)

Where *L*_*i*_ represents the local *Moran’s I*, and the other variables are the same as above.

### 2.3 Factors affecting enterprise innovation

The influencing factors of SIDRC are diverse, and this study mainly discusses from four aspects: policy support index (PSI), economic development level (GDP), sports industry structure diversity (SISD) and social participation index (SPI). Specifically, the policy support index (PSI) reflects the government’s support for the sports industry and is an important driving force to promote the development of the sports industry. This index is calculated by the entropy weight method from the number of sports laws and regulations, the number of non-governmental sports organizations, the number of sports advertisements and the number of texts related to the sports industry in government reports. The level of economic development (GDP) reflects the economic strength of a region and has an important impact on the consumption demand and investment willingness of the sports industry. The structural diversity of sports industry (SISD) measures the diversity and innovative development ability of sports industry, which is very important for dispersing external risks and improving industrial competitiveness. Social Participation Index (SPI) shows the public’s participation in sports, sports infrastructure coverage and sports culture atmosphere, and reflects the social support and demand for sports industry. Commonly used spatial econometric models include spatial lag model, spatial error model and spatial Dobbin model.

(1) The spatial lag model is a spatial autoregressive process, and its basic expression is:

$$\text{ln}\;SIDRC_{i,t}=\rho W\;\text{ln}\;SIDRC_{j,t}+\beta_{i}X_{i}+\mu_{i}+\eta_{i}+\varepsilon_{i,t}$$ (9)

Among them, ln *SIDRC*_*i*,*t*_ is the logarithm of the toughness index of sports industry development, *W* is the spatial weight matrix, *ρ* is the spatial lag parameter, and *μ* is the white noise interference term.

(2) The spatial error model explains the spatial correlation and heteroscedasticity between variables, and the specific form is:

$$\begin{array}{c} \text{ln}\;SIDRC_{i,t}=\beta_{i}X_{i}+\mu_{i}+\eta_{i}+\varphi_{i,t}\\ \varphi_{i,t}=\lambda W\varphi_{i,t}+\varepsilon_{i,t} \end{array}$$ (10)

Where *ε* represents regression residual vector, *λ* is autoregressive coefficient, and other variables are consistent.

(3) The spatial dobbin model is a synthesis of spatial lag model and spatial error model, and its specific form is:

$$\text{ln}\;SIDRC_{i,t}=\rho W\;\text{ln}\;SIDRC_{j,t}+\beta_{i}X_{i}+WX_{i,t}\gamma +\mu_{i}+\eta_{i}+\varphi_{i,t}$$ (11)

Among them, *γ* is the coefficient of interaction between linear independent variable and spatial lag dependent variable, which reflects the spatial spillover effect.

### 2.4 Index system

Resilience theory has broad application in describing and explaining the coping abilities of complex systems, such as individuals, organizations, and societies, and can help decision-makers determine when to change operational modes or development strategies to cope with future uncertainties. This study borrows from resilience theory to propose the concept of a resilience index for the development of the sports industry, which mainly embodies three aspects: adaptive capacity, self-organizing ability, and self-recovery ability. Adaptive capacity reflects the ability of the sports industry to maintain stable development in the face of external shocks. For example, during the COVID-19 pandemic, the sports industry can expand its business scope through online marketing and events to maintain stable development. Self-organizing ability represents the macro manifestation of the market-oriented, commercialized, and standardized development of the sports industry, implying that the industry structure is reasonable, and the production chain, sales chain, and consumption chain are coupled and coordinated, forming a virtuous cycle of industry development. Self-recovery ability reveals the ability of industry development to gradually recover to a stable and orderly state after being affected by external influences and experiencing stagnation or setbacks. As the world begins to enter the post-pandemic era, the sports industry is also entering a period of revival and gradually recovering its vitality.

Based on the aforementioned analysis, it can be seen that the resilience index for the development of the sports industry involves a broader scope compared to indicators such as competitiveness and high-quality index, and its evaluation indicators also need to follow basic principles of comprehensiveness and scientific rigor. Therefore, this study constructs an evaluation index system for the resilience index of the sports industry development from four dimensions: input and output, structure and innovation, market and consumption, and society and policy (Table 1). Specifically, the input and output dimension includes the basic elements and fundamental goals of sports industry development, which directly impacts the industry’s development vitality and quality and reflects the operating status and market performance within the industry. The structure and innovation dimension reveals the key factors for achieving sustainable development in the sports industry, where industry structure rationality and technological innovation capability play a crucial role in enhancing future industry competitiveness. The market and consumption dimension are the most significant and basic characteristics of the sports industry, where indicators of consumption level and market size reflect the development potential of the sports consumption market, which plays an important role in enhancing industry self-organizing ability. The society and policy dimension represents the basic environment in which the sports industry develops and has a significant regulating effect on its development quality and effect, while providing institutional guarantees and material basis for enhancing the industry’s resilience.

**Table 1:** SIDRC evaluation index system.

Target layer	Criterion layer	Index layer	Weight
SIDRC	Input and output (0.3007)	Total investment in sports industry(Kim et al., 2023)	0.0353
		Total financing of sports industry(Liu et al., 2023)	0.0321
		Number and scale of sports funds(Author selection)	0.0580
		Number of sports personnel and coaches(Gupta et al., 2022)	0.0209
		Gross output value of sports industry(Author selection)	0.0527
		Added value of sports industry(Kim et al., 2023)	0.0479
		Total number of people employed in sports industry(Liu et al., 2023)	0.0538
	Structure and innovation (0.2439)	Proportion of output value of sports service industry(Author selection)	0.0514
		Proportion of export output value of sporting goods(Bellver et al., 2022)	0.0370
		Investment in scientific R&D of sports industry(Author selection)	0.0572
		Number of sports enterprises(Author selection)	0.0489
		Total sports technical achievements(Liao et al., 2021)	0.0494
	Market and consumption (0.1627)	Consumption level of sports market(Jin et al., 2023)	0.0379
		Market scale of sports industry(Author selection)	0.0412
		Average income of sports employees(Liu et al., 2023)	0.0448
		Growth rate of sports consumption market(Author selection)	0.0388
	Society and policy (0.2927)	Sports health and knowledge popularization rate(Kim et al., 2023)	0.0560
		Supply of sports public facilities(Author selection)	0.0541
		Per capita sports facilities area(Author selection)	0.0308
		Growth rate of sports infrastructure(Author selection)	0.0521
		Total government investment in sports industry(Liao et al., 2021)	0.0705
		Number of government sports special documents(Wang et al.,2019)	0.0292

### 2.5 Data source

Considering the availability of data and comparability of results, this study focuses on 285 cities in China from 2010 to 2022. During this period, the sports industry in China has developed rapidly and formed a good industrial structure, which not only provides valuable reference for the development of sports industry in other developing countries, but also provides empirical basis for guiding the future industrial layout and direction. The data used in this study are from the National Bureau of Statistics (http://www.stats.gov.cn.2023.04), China Sports Industry Development Report, China City Statistical Yearbook and Social Science Database (http://www.ppm anda.cn.2023.04). After obtaining the initial data set, SPSS software is used to clean the data, remove the abnormal values and supplement the missing values by linear interpolation. According to Formula 1–4, the development toughness index of sports industry is calculated, and the calculation results are statistically analyzed with the help of state software to master the data distribution structure. At the same time, the city-scale administrative divisions of China were obtained from the resource and environmental science data registration and publishing system (https://www.resdc.cn/DOI/), and the calculation results were visualized by ArcGIS software on the basis of the map, and whether there was spatial correlation was tested.

## 3. Empirical analysis

### 3.1 Temporal distribution characteristics analysis of IDRC

**Fig 1:**
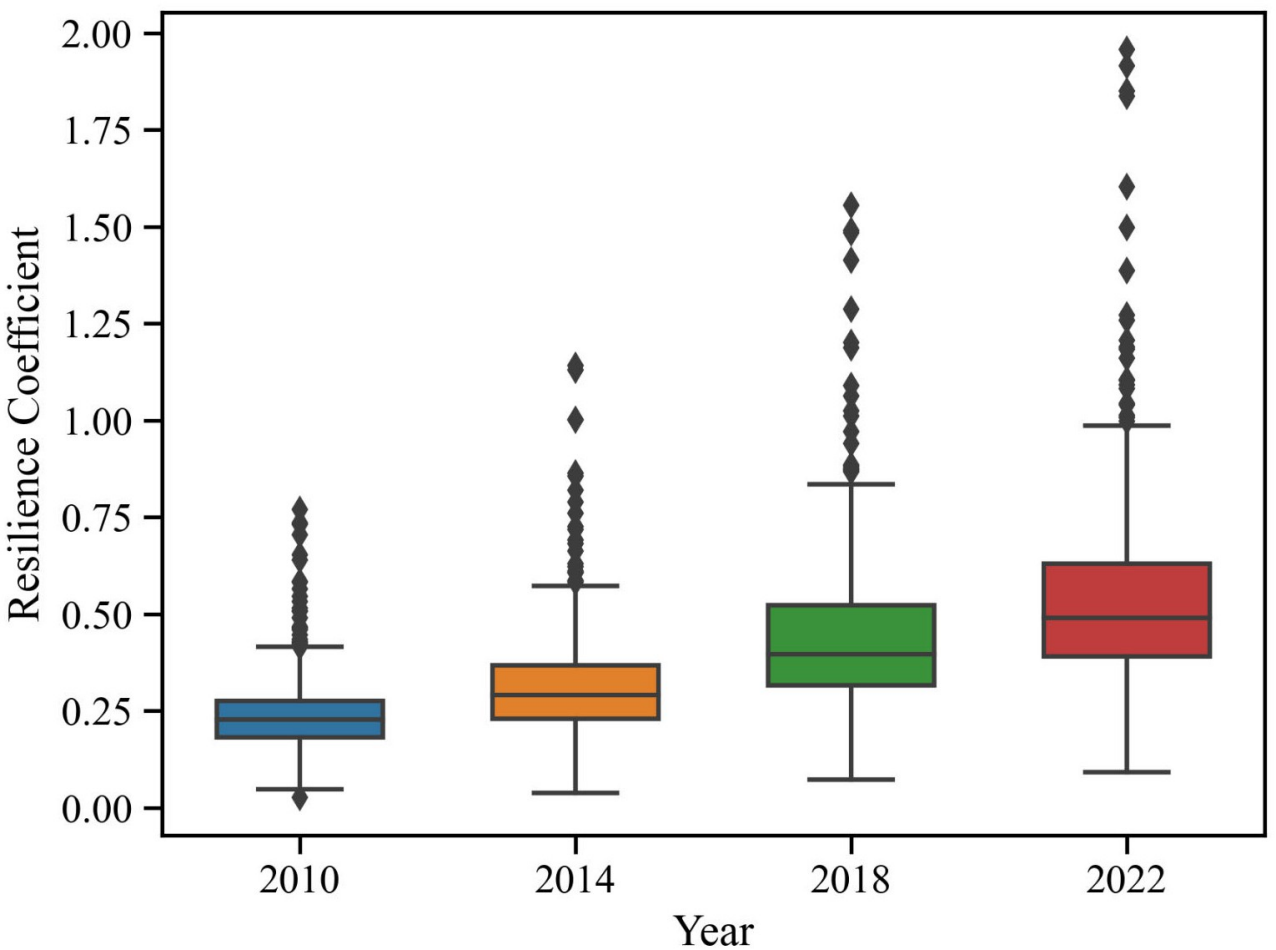
Box diagram of SIDRC in different years.

Based on Eqs 1–4, the resilience index of the sports industry development in China from 2010 to 2022 was calculated, and the sample structure was organized using PyCharm software. Fig 1 shows box plots of the resilience coefficients of the sports industry development in 285 Chinese cities for the years 2010, 2014, 2018, and 2022. The median line represents the median resilience coefficient of different years, while the box, consisting of the first quartile (Q1) and the third quartile (Q3), reveals the key part of the sample structure of the resilience coefficient. The whiskers represent the sample values outside Q1 and Q3, and their length reflects the sample structure’s degree of dispersion. Specifically, the median line was around 0.25 in 2010, and the box was relatively short, indicating that the resilience coefficients of the sports industry development in Chinese cities were at a generally low level, with no significant regional differences. With the passage of time, the median line slowly rose to around 0.5 in 2022, and the box gradually became higher and the whiskers extended to both sides. This change indicates that although the resilience coefficients of the sports industry development showed growth throughout the entire study period, the overall resilience level remained relatively low, and the gaps between cities were gradually widening. It is particularly noteworthy that there are still many outliers outside the whiskers, and their distances are gradually increasing, revealing the potential risk of unbalanced development in the sports industry. In other words, similar to the digital divide produced by the digital economy, China’s sports industry development may fall into the "Matthew Effect" trap, where high-resilience cities are more likely to obtain high-quality sports industry resources (facilities, technologies, talents, etc.) due to the siphon effect, leading to low-resilience cities losing competitive advantages and forming a vicious cycle of cumulative growth, resulting in increasingly significant gaps between cities.

In order to further explore the data distribution characteristics of SIDRC, this study makes descriptive statistics on the calculation results (Table 2). It can be found that the variance increased from 0.013 in 2010 to 0.082 in 2022, and the standard deviation increased from 0.116 in 2010 to 0.288 in 2022, which means that the interval SIDRC gap increased and the data dispersion characteristics began to strengthen. Although the growth rate is small, combined with Fig 1, it can be found that more and more cities begin to grow rapidly, that is, the gap between regions begins to increase gradually, which is unfavorable to the cross-regional flow of sports resources.

**Table 2:** Descriptive statistics of SIDRC.

Year	Max	Min	Interpolation	St.d	Var
2010	0.770	0.026	0.744	0.116	0.013
2014	1.142	0.038	1.104	0.166	0.028
2018	1.555	0.073	1.482	0.233	0.054
2022	1.957	0.092	1.865	0.288	0.082

### 3.2 Spatial distribution pattern analysis of SIDRC

**Fig 2:**
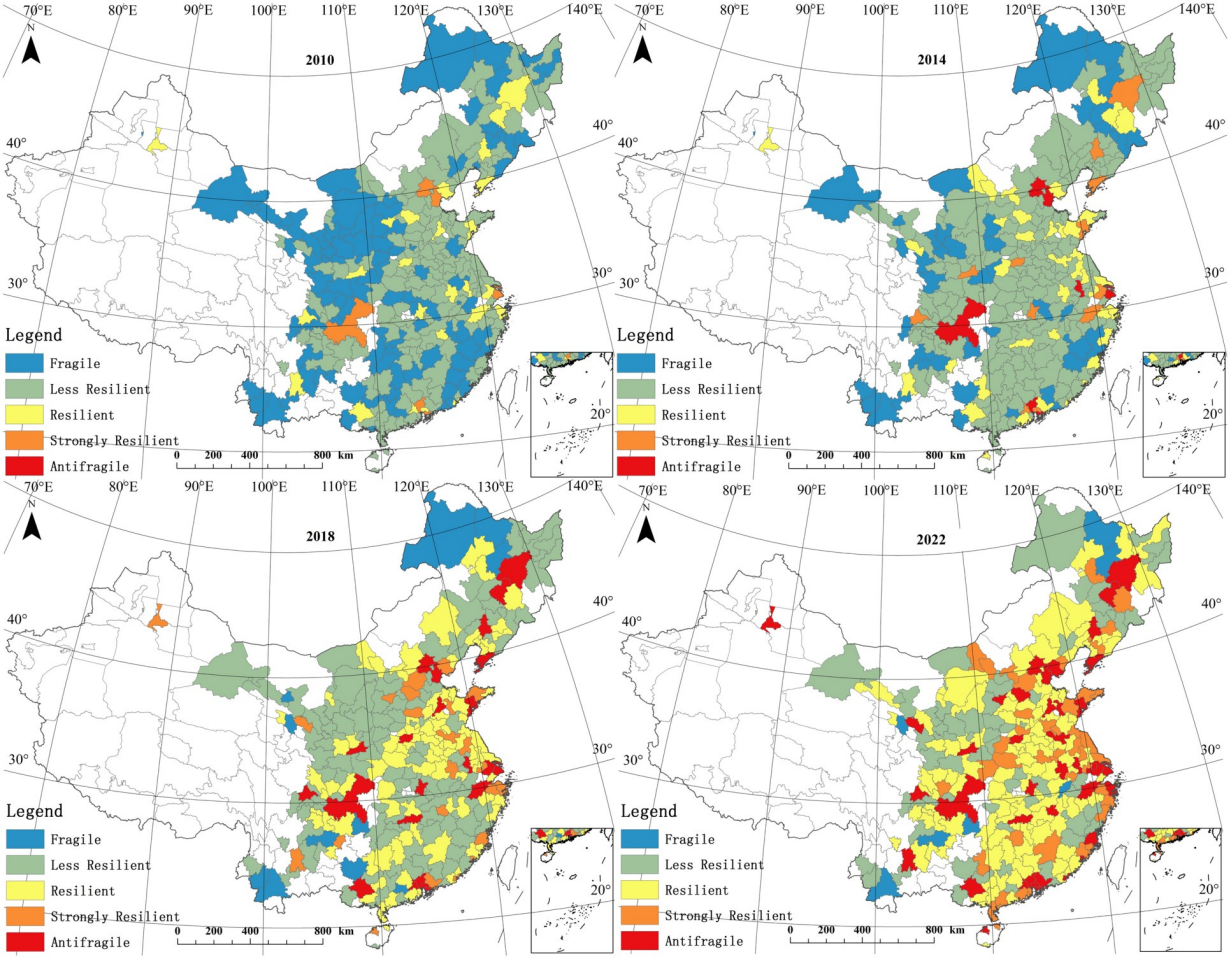
Spatial distribution pattern of SIDRC (the administrative boundaries of prefecture-level city are republished from [16] under a CC BY license, with permission from the resource and environment data center, original copyright 2022).

The ArcGIS 10.2 software was utilized for the visualization of the SIDRCin China from 2010 to 2022. To facilitate comparability, the resilience coefficient was segmented into five categories. When *SIDRC* ∈ (0, 0.2], the sports industry is assigned to the Fragile group. This implies that the sector is vulnerable to external impacts, has limited adaptability, is small in scale, and is incapable of quickly resolving the external effects. When *SIDRC* ∈ (0.2, 0.4], the sports industry belongs to the Less Resilient group. Such industries are sensitive to external impacts but possess significant recovery and adaptability capabilities. A *SIDRC* ∈ (0.4, 0.6] is designated to the Resilient group, indicating that the sports industry can promptly adjust to external impacts, maintain basic operations, and reduce the impact of external pressures through risk management strategies and process optimization. A *SIDRC* ∈ (0.6, 0.8] denotes the Strongly Resilient group, in which the sports industry can maintain growth and competitive advantages through innovation and private enterprise and make progress during external impacts. A *SIDRC* ∈ (0.8, 1] places the industry under the Antifragile group, signifying that it can quickly adapt, grow, and enhance its adaptability in intricate and uncertain external environments. Additionally, the higher the external impact, the greater the industry’s innovation and growth capabilities. Fig 2 illustrates the spatial distribution of China’s SIDRC at different time intervals.

The SIDRC considerably improved during the study period. In 2010, 102 and 152 cities were classified as belonging to the Fragile and Less Resilient groups, respectively, accounting for 89.123% of the total sample. Additionally, only six cities, namely Beijing, Tianjin, Guangzhou, Shenzhen, Chongqing, and Shanghai, attained the Strongly Resilient level, and no city reached the Actifragile level. At this period, China’s sports industry had a weaker foundation for development, making it less adaptable to external impact. In terms of sports infrastructure investment, product innovation, and market construction, there remained a gap compared to highly developed countries such as the United States and Japan. By 2022, China’s sports industry SIDRC had improved impressively. The number of cities that belonged to the Less Resilient group decreased by 53.947% compared to 2010. Meanwhile, the number of cities in the Resilient and Strongly Resilient groups increased by 99 (a 396% rise) and 38 (a 633.333% rise), respectively. This implies that these two levels of cities will play a critical role in reducing the regional differences in SIDRC, becoming a powerful driving force. Furthermore, 40 cities progressed into the Antifragile group, indicating that they possess a more stable and robust sports industry and market. Nevertheless, despite the conspicuous increase in each level’s number of cities, the significant issue of sample structure imbalance displayed in Fig 1 still persisted. Specifically, many cities still carried highly fragile SIDRC in the sports industry, such as Jincheng, Qingyang, and Shangluo. These cities possess a disadvantage in terms of infrastructure investment, consumer market, and policy guidance, and, as such, risk becoming the periphery regions for balanced SIDRC development.

The uneven distribution of SIDRC in China is reflected in two aspects: sample structure and spatial pattern. The former was explained in the previous section, and here we focus on spatial unevenness. Cities ranked as Fragile and Less Resilient dominated the geographical space in 2010, with the former forming a "block-shaped" spatial pattern in regions such as northeastern Inner Mongolia, Gansu Province and Ningxia, southern Yunnan Province, and parts of Hunan Province and Jiangxi Province. By 2014, these "block-shaped" regions were further divided, and Less Resilient became the main distribution type, with cities such as Chongqing, Beijing, and Tianjin entering the Antifragile level. In 2018, the Resilient level began to appear heavily, indicating that the Chinese sports industry had entered a new stage, with significant improvements in industry structure, infrastructure layout, and sports consumer market, and an increase in the capacity for innovative development of the industry. The spatial pattern in the form of an interlocked distribution with Less Resilient was observed. Until 2022, Strongly Resilient and Antifragile began to appear heavily, and the Resilient level became dominant. Overall, the spatial evolution of SIDRC in China’s sports industry went through four stages: Fragile and Less Resilient interlocking distribution-Less Resilient balanced distribution-Less Resilient and Resilient interlocking distribution-Resilient and Antifragile interlocking distribution. However, there are still regional and urban disparities, with Eastern regions and provincial capital cities remaining superior.

### 3.3 Spatial correlation analysis of SIDRC

**Fig 3:**
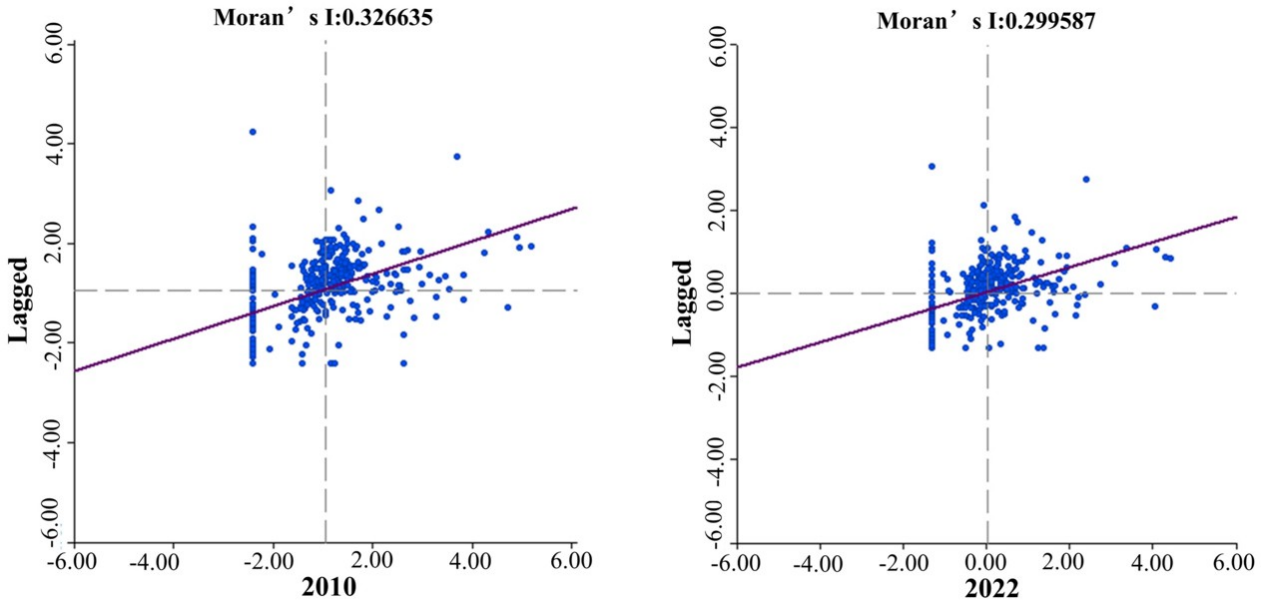
Local spatial correlation of SIDRC.

This study further investigates whether there is spatial correlation in the SIDRC at the local level. Fig 3 shows the scatter plot of the distribution of SIDRC under inverse distance weighting. H-H indicates that the SIDRC of a city and its surrounding cities are both at a higher level, which results in an anti-fragile region. H-L indicates that a city has a strong resilience while the SIDRC of adjacent cities is at a fragile level, resulting in a spill-over effect. Similarly, L-H indicates that a city has weak resilience while the SIDRC of surrounding cities is at an anti-fragile level, resulting in a chasing effect. L-L indicates that both a city and its surrounding cities are at a fragile level, resulting in a fragile region.

Calculated based on Eqs 4–5, the global Moran’s I index during the study period were 0.153, 0.159, 0.173, and 0.181, respectively, indicating a positive spatial correlation in the SIDRC of the sports industry. This means that the spatial distribution is not random, and cities with similar SIDRC are more likely to cluster. When the external environment changes, cities with similar or the same SIDRC are more likely to form complementary relationships in terms of resources, knowledge, and other factors. The local Moran’s I index decreased from 0.326635 to 0.299587, indicating a gradual decline in local correlation. This may be due to the development of information technology and digital economy, which compresses the spatial and temporal effects of sports industry resources and market size. The spill-over effect of highly resilient cities has a wider impact range.

**Fig 4:**
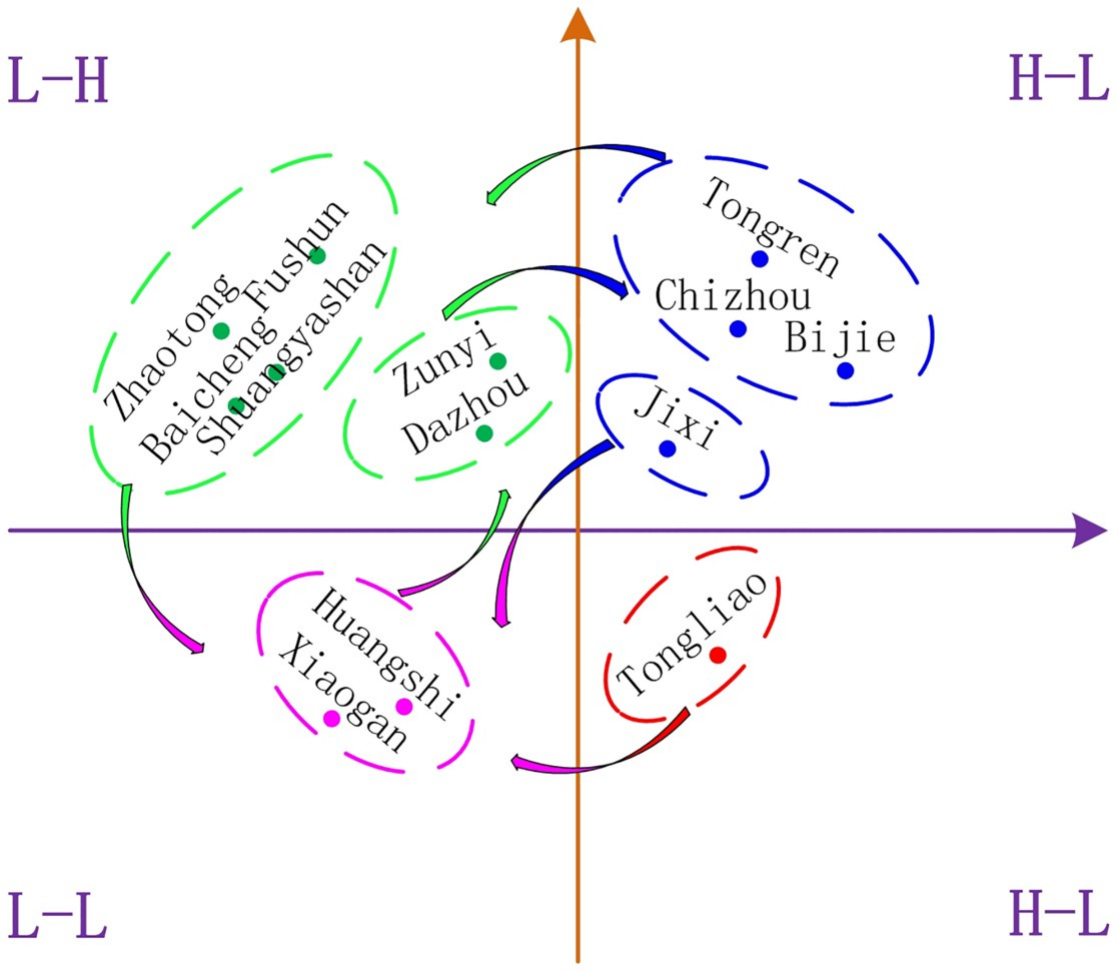
Time-space transition of SIDRC.

Compared with the scatter plot distribution in 2010 and 2022, some cities have experienced quadrant changes, known as “leapfrogging,” at different times, revealing the local changes of the SIDRC. Fig 4 shows the spatiotemporal leapfrogging process of SIDRC. Specifically, Zunyi and Dazhou leapt from the L-H quadrant to H-H, known as “upstream leapfrogging,” reflecting the process of the city’s SIDRC changing from fragility to resilience. Zhaotong and Fushun, among others, leapt from L-H to L-L, while Huangshi and Xiaogan leapt from L-L to L-H, and Jixi leapt from H-H to L-L, known as “downstream leapfrogging,” describing the process of the city’s SIDRC transforming from resilience to fragility. Overall, downstream leapfrogging is dominant, and the number of leapfrogging instances is relatively small, indicating a relatively stable spatiotemporal evolution of the SIDRC.

### 3.4. Influence factors analysis of SIDRC

#### 3.4.1. Result analysis

Different types of spatial econometric models can be transformed into one another. To obtain a better fit, this study employs the Wald test and the likelihood ratio test to select the model. The results pass the 1% significance level, indicating that the spatial Durbin model does not suffer from degeneracy. Combining the adjusted R-squared, results show that the SDM model has the best fit. Therefore, this study analyzes the data based on this model. The specific regression results are shown in Table 3.

**Table 3:** Spatial econometric regression results.

Variable	OLS	SAR	SEM	SDM
ln *PSI*	0.012*	0.132*	0.425*	0.643**
ln *GDP*	-0.021	0.315***	0.614***	1.321***
ln *SISD*	0.015	2.655	0.153	0.794***
ln *SPI*	0.032	-0.014	-0.215	1.532***
ln *PSI* * *W*	-	-	-	0.890
ln *GDP* * *W*	-	-	-	-0.031*
ln *SISD* * *W*	-	-	-	0.215
ln *SPI* * *W*	-	-	-	0.912**
*R*^*2*^—*ad*	0.431	0.510	0.631	0.723
*Lag − likehood*	-	456.63	589.12	715.32
Individual effect	Control	Control	Control	Control
Time effect	Control	Control	Control	Control
Sample size	3420	3420	3420	3420

The regression coefficient between the policy support index and the SIDRC is 0.643, which is significant at a 5% confidence level. Thus, policy support plays a significant role in promoting the resilience of the sports industry. On one hand, the government formulates corresponding policies that create a favorable business environment for sports enterprises, such as tax reductions, financial support, optimized market access mechanisms, and the reduction of production and operation costs. Additionally, the government’s industrial planning and development strategies provide direction for promoting the convergence and allocation efficiency of different types of sports resources, which promotes the transformation and upgrading of the industry’s structure. On the other hand, policy support stimulates the innovation capacity of the sports industry and the spread of sports culture. Sports enterprises realize digital transformation by developing new technologies, products, and services, enhancing the competitiveness, adaptability, and resilience of the sector. The popularization and spread of sports culture have also increased public awareness and understanding of the industry, promoting the public’s demand for sports market and spiritual demand of sports culture, resulting in improving the toughness of industrial development. Finally, the spatial lag coefficient of the policy support index is 0.890, which is not significant, demonstrating that the improvement of the policy support index in one city will not have a significant impact on the SIDRC of neighboring cities.

The regression coefficient of the level of economic development on SIDRC is 1.321, and it is significant at the 1% confidence level, indicating that economic development has a significant positive effect on enhancing the resilience of the sports industry. In other words, when the level of economic development increases by 1%, it can promote a 1.321% increase in SIDRC. Combining with the previous text, it can be found that the SIDRC in China’s eastern coastal areas and provincial capital cities is generally higher than that in other cities, which can be partially explained by the difference in the level of economic development. Cities with high levels of economic development usually have higher residents’ income levels and consumption capabilities, which also lead to the growth and expansion of the sports market demand for sports products, infrastructure, sports events, and services, etc. The benign cumulative effect of market demand and industry resilience enhancement helps to achieve sustainable development of the sports industry. In addition, regions with high levels of economic development often have richer educational resources and talent reserves, providing good resource supply for the development of the sports industry and enhancing its adaptability to external shocks. The spatial lag coefficient of the level of economic development is -0.031, and it is significant at the 10% confidence level, indicating that the level of economic development will have a negative effect on the promotion of SIDRC in surrounding cities. One possible reason is that developed cities generate a siphon effect, resulting in the loss of advantageous resources such as talents, technology, and enterprises in neighboring cities, thereby inhibiting the promotion of SIDRC.

The regression coefficient of the diversity of sports industry structures on SIDRC is 0.794, and it is significant at the 1% confidence level, indicating that the diversity of industry structures has a positive effect on strengthening the local external shocks of the sports industry. The diversity of sports industry structures means diversified development fields and formats, such as sports equipment manufacturing, sports event organization, sports venue construction, sports training, etc. These sub-industries can not only generate more new areas combined with the current digital transformation and green development but also help to disperse and avoid risks by changing the development mode when affected by external influences. Moreover, diversified industry structures also facilitate communication and cooperation between various fields, promote the generation of innovative thinking, and achieve the effect of 1+1>2. The spatial lag coefficient of the diversity of sports industry structures is 0.215, but it is not significant, indicating that it does not have a significant impact on the promotion of SIDRC in neighboring areas.

The regression coefficient of the social participation index on the SIDRC is 1.532, and it is significant at the 1% confidence level, indicating that it has a significant promoting effect on enhancing the resilience of the sports industry. On the one hand, areas with high social participation have higher attention and participation in the field of sports, making it easier to construct and disseminate sports culture and establish more branded sports products or organizations. Brand effects help to create higher visibility and occupy more market share in the sports industry. On the other hand, social participation brings more extensive financing channels. Due to people’s attention to the development of the sports industry, relevant enterprises are more likely to obtain financing and expand the market. The support of financial institutions, investors, and sponsors reduces the production costs of the sports industry. The spatial lag coefficient of the social participation index is 0.912, and it is significant at the 5% confidence level, indicating that it has a positive effect on the SIDRC in neighboring cities. This spillover effect may come from three aspects: resource sharing, market expansion, and extension of the industry chain. Urban agglomeration has become a new type of economic agglomeration, providing a foundation for sharing sports resources or facilities among cities. When the sports industry development resilience of a city is relatively high, its surrounding cities may benefit from resource or facility sharing, which has a positive effect on the development of their own sports industry. The growth of market demand may affect neighboring cities, bringing more market opportunities and helping to improve the SIDRC in neighboring areas. The location advantages between neighboring cities help to extend the sports industry chain, to some extent, promoting the improvement of the SIDRC in neighboring areas.

#### 3.4.2. Robustness test

The research chooses two methods to test the robustness of the SDM model: delaying the SIDRC by one period and shortening the time window. Table 4 describes the parameter changes of the model under the two test methods. It can be found that in both SDM-lag model and SDM-time model, the sign of each variable is changed, which shows that the original model is robust.

**Table 4:** Robustness test result.

Variable	SDM-lag	SDM-time
ln *PSI*	0.311*	0.563**
ln *GDP*	0.214**	0.912***
ln *SISD*	0.313*	0.884**
ln *SPI*	-0.105	1.312***
ln *PSI* * *W*	-	0.635
ln *GDP* * *W*	-	-0.101*
ln *SISD* * *W*	-	0.118
ln *SPI* * *W*	-	0.382**
*R*^*2*^—*ad*	0.515	0.235
*Lag − likehood*	389.12	637.32
Individual effect	Control	Control
Time effect	Control	Control
Sample size	3420	3420

## 4. Policy implications

In view of the above research, this paper puts forward some suggestions from four aspects: policy formulation, market construction, industrial structure adjustment and public participation:

1. Strengthen the policy guidance to the sports industry, improve the pertinence and flexibility of relevant policies, and formulate corresponding policies according to the characteristics of different regions and markets. For example, in view of the relatively weak development of sports industry in the central and western regions of China, we can increase the intensity of tax relief, financial subsidies, land and other resources allocation, create an excellent investment and entrepreneurial environment, and attract more investors and enterprises to enter the sports industry. At the same time, the government should strengthen the training and introduction of sports talents to improve their competitiveness.
2. Increase investment in sports market construction, improve market infrastructure construction, and improve the competitiveness and attractiveness of sports industry market. Especially in the process of building new urbanization and sports parks, we should strengthen the construction of supporting facilities and increase the investment in the integration of intelligent technology and venue facilities. At the same time, encourage enterprises to innovate and brand, better meet the market and consumer demand, and improve the quality of products and services.
3. Adjust the industrial structure reasonably according to the market demand, talents and resource advantages. While focusing on the development of traditional industries such as sports goods manufacturing, sports product sales and sports services, we should encourage the sports industry to accelerate the digital transformation, integrate information technology with the traditional sports industry, and promote the digital and intelligent development of the sports industry. In addition, pay attention to the role of pilot demonstration, and gradually strengthen the brand building and market competitiveness of small and medium-sized sports brands through industrial chain cooperation and policy promotion, and appropriately optimize the resource allocation between regions and markets.
4. Improve the public’s awareness and enthusiasm for participating in sports, increase the opportunities for the public to participate in sports activities, and improve the sustainability of the sports industry. Increase investment in public services such as sports events, training facilities and sports public welfare, strengthen the connection between basic units such as families, schools and communities and the sports industry, and gradually promote the popularization of sports culture from the basic level of society. At the same time, create a good sports atmosphere and sports culture atmosphere, and improve the importance and development potential of sports in the whole society.

## 5. Conclusion and discussion

### 5.1 Conclusion

This study evaluates and visualizes the resilience of the sports industry development in 285 cities in China using the Topsis model and exploratory spatial data analysis. We also construct a spatial econometric model to discuss the influencing factors of the sports industry SIDRC. The main conclusions are as follows:

1. The SIDRC of the Chinese sports industry has significantly improved, but the growth rate is relatively slow, and overall, it still remains at a low level of resilience. The Matthew effect has caused the gap between cities to widen further. Cities classified as Resilient or Strongly Resilient play a critical role in narrowing the regional SIDRC gap, while cities such as Jincheng, Qingyang, and Shangluo have consistently exhibited high vulnerability and serve as the outskirts of balanced development.
2. The Chinese sports industry SIDRC exhibits a significant spatial disequilibrium, with Fragile and Less Resilient cities dominating early research and presenting a "block-like" distribution pattern in space. With time, Resilient cities have dominated, but the distribution pattern has consistently favored the coastal eastern region over the central and western regions, and provincial capitals over other cities. The sports industry SIDRC displays strong spatial positive correlation, with relatively low local spatial variation rates.
3. The Policy Support Index, economic development level, diversity of sports industry structure, and social participation all have a significant promoting effect on the sports industry SIDRC. High economic development level cities cause obstacles to the development of the SIDRC of the sports industry in neighboring cities due to the "siphon effect’’ that causes talent and resource outflows. Social participation promotes the resilience enhancement of neighboring cities through resource sharing, market expansion, and the extension of the industry chain.

### 5.2 Discussion

The purpose of this study is to evaluate the resilience of sports industry development, identify its temporal and spatial differences, and explore its influencing factors. It is found that the development resilience of sports industry in China is rising continuously, and the growth rate is relatively slow, which is similar to the research conclusion of (Moretti et al., 2023). In addition, this study also found that there are significant spatial heterogeneity and spatial agglomeration in the development resilience of sports industry, and (Kin et al., 2023) also put forward similar views in the study, but in comparison, the index system of this study is more perfect. This study found that social participation index has a positive effect on the development resilience of sports industry, but some related studies put forward the opposite view, which may be due to the different variable sets selected by the regression model, and there is a regulatory effect between variables. Therefore, this study believes that there are still the following aspects that can be further improved in the follow-up work. (1) The spatial correlation of the sports industry SIDRC discussed in previous sections can be abstracted as a related network, or the development related network of the sports industry, which reveals the regional adaptability and resilience from the aspects of correlation and integrity, and has more connotation than the current study from the perspective of "flow space’’ theory; (2) The influencing factors of the sports industry SIDRC selection in this study mostly focus on social and economic aspects, which is clearly not sufficient and should be further explored in future research. These problems will be discussed further in our subsequent research.
